# Workplace coaching: a meta-analysis and recommendations for advancing the *science of coaching*

**DOI:** 10.3389/fpsyg.2023.1204166

**Published:** 2023-10-10

**Authors:** Janis A. Cannon-Bowers, Clint A. Bowers, Catherine E. Carlson, Shannon L. Doherty, Jocelyne Evans, Julie Hall

**Affiliations:** ^1^Cannon-Bowers Consulting, Apopka, FL, United States; ^2^Department of Psychology, University of Central Florida, Orlando, FL, United States

**Keywords:** workplace coaching, executive coaching, coaching, leadership development, coaching effectiveness

## Abstract

Workplace coaching has experienced a dramatic rise in popularity over the past decade and is one of the fastest growing performance-enhancing interventions used by modern organizations. Yet, despite its popularity, workplace coaching has not been the subject of much empirical research and a true *science of coaching* has yet to be developed. The purpose of this research was to update prior meta-analyzes that investigated the impact of coaching on organizational outcomes and to provide recommendations for how the field needs to evolve. Results indicated that, consistent with prior meta-analyzes, workplace coaching is effective in achieving positive organizational outcomes. The effects of several moderators were also investigated. Finally, we discuss the results in terms of recommendations for future directions that we believe will establish and advance the *science of coaching*.

## 1. Introduction

Coaching has experienced a dramatic increase in interest and use in the past several years. In fact, coaching has been described as one of the fastest-growing specialties within the Human Resources profession (Bozer and Delegach, 2019). The International Coaching Federation (ICF) reported that there were more than 71,000 coaching professionals in 2019 (International Coaching Federation, 2020), a number that has more than tripled in the past 10 or so years (Theeboom et al., 2014). Indeed, coaching in the workplace has been so well accepted that many organizations provide it as part of a benefit package to their most valued employees. The ICF estimates that over two billion US dollars per year is invested in workplace coaching worldwide (International Coaching Federation (ICF), 2020).

As coaching has increased in acceptance, it has also evolved to meet the demands of its clients. Coaches are much more likely to have received formal training now than in the past (Passmore and Sinclair, 2020). There are also an increased number of assessment techniques (Möeller and Kotte, 2022) and interventions (Greif et al., 2022) available to today’s coaches. Additionally, the COVID pandemic accelerated the shift in the delivery modality of coaching, with many coaches switching to technology-based delivery platforms rather than face-to-face interactions.

Despite the popularity of workplace coaching in practice, scientists have lamented the lack of empirical research in this area (Jones et al., 2016; Silzer et al., 2016). Critics have questioned whether coaching is actually empirically based (Sherman and Freas, 2004; Greif et al., 2022) or worth level of investment (Sonesh et al., 2015). Fortunately, however, researchers have begun to respond to this need, with a dramatic uptick in research examining coaching effectiveness over the past decade (Kotte and Bozer, 2022).

This increase in research activity, combined with the rise in popularity of workplace coaching, drive the need for another review of the scientific literature to allow us to assess the state of the art and to suggest directions for future research. Hence, the present manuscript describes a meta-analytic review of research on the effectiveness of workplace coaching since 2018. This review also considers the impact of several moderator variables that may influence the effectiveness of coaching.

Ultimately, our goal is to provide a set of prescriptions that will move the field toward a true *science of coaching*. At present, the field of coaching is still relatively immature (albeit very popular) in the sense that we do not understand exactly what works, what the underlying mechanisms of action are, which coaching approaches are most effective, or how long coaching needs to take to achieve results. Our analyzes and subsequent discussion and conclusions will attempt to address these questions.

### 1.1. Definition of coaching

Workplace coaching (which includes executive coaching and other coaching interventions aimed at improving performance in the workplace) is defined in several ways in the literature (see Greif et al., 2022 for a review). Some of these definitions incorporate the idea that coaching is a counseling and support process (Greif et al., 2022), while others emphasize goal setting and organizational outcomes (Kilburg, 1996; Grant, 2003). Perhaps the most accepted definition of coaching is that of the International Coaching Federation, which defines coaching as, “Partnering with clients in a thought-provoking and creative process that inspires them to maximize their personal and professional potential” (International Coaching Federation, 2020). Notably, what most of these definitions have in common is the notion that coaching involves an intimate relationship between a coach and a client (or group of clients) that is aimed at improving the client’s outcomes, the organization’s outcomes or both. In this sense, there is a fairly wide consensus around what executive coaching is meant to accomplish.

Where the definitions diverge somewhat, is in how they define the coaching process itself. Indeed, coaching has been described using a wide variety of words such as: counseling, behavior modification, facilitation, appreciative inquiry, problem solving, goal setting, etc. (Greif et al., 2022). Unfortunately, this conceptual confusion and lack of specificity around the exact nature of the coaching process makes it difficult to evaluate coaching research, to compare outcomes across coaching studies or to provide guidance for training new coaches. Moreover, if coaching is defined as any type of interaction between coach and client, with few documented strategies, approaches, tools or prescriptions unacceptable variability in outcomes is to be expected. That means that some coaching situations succeed better than others for reasons that cannot be easily discerned.

While it is unlikely that a single, agreed upon definition of coaching will be accepted any time soon, one way to begin to establish parameters around the various approaches to coaching is to understand the “mechanisms of action.” That is, we need to better understand the theoretical justification for why various coaching approaches are expected to achieve desired outcomes. To date, several theoretical bases have been proposed to guide coaching practice. These are reviewed in the following section.

### 1.2. Theoretical bases of coaching

In general, coaching practice has been heavily influenced by Positive Psychology which focuses on positive aspects of human experience (as opposed to mental illness or maladaptive behavior). Indeed, according to Auer et al. (2022), many have suggested that “coaching can be thought of as an applied form of positive psychology (Grant and Cavanagh, 2007, p. 3) or that coaching fits appropriately within the broader positive psychology framework (Freire, 2013; Theeboom et al., 2014). Hence, the influence of positive psychology is evident across coaching approaches.

That said, prevailing reviews of coaching generally converge on two different theoretical bases upon which coaching practice is defined (Bono et al., 2009; Vandaveer et al., 2016). On the one hand, coaching has been conceived as primarily a facilitation process that has its roots directly in positive psychology and includes techniques such as appreciative inquiry and counseling. The emphasis from this perspective is on the *process* of coaching (Williams and Lowman, 2018). That is, the coach’s role is to provide active and empathic listening, Socratic questioning, and clarification with the aim being to help the client remove barriers that are keeping them from achieving their personal and professional goals (Vandaveer et al., 2016). It is largely non-directive and aimed at helping the client gain insights and actualize their potential.

The second view of coaching puts the emphasis more squarely on the *outcome* of coaching by focusing on goal setting and goal achievement (Whitmore, 2010). The theoretical basis for this approach rests on literature into goal setting, including action planning and accountability as a means to achieve durable behavioral change. Some of the specific approaches that fall into this category include strength-based coaching (MacKie, 2014) and Cognitive-Behavioral coaching (Passmore et al., 2013). The common ingredient is that the coach’s role is to help the client clearly define their goal, develop concrete actions plans designed to achieve the goal and set up mechanisms so that the client is accountable for their progress towards achieving the goal (Grant, 2022).

As noted, the influence of positive psychology is evident in these approaches as well. For example, strength-based coaching focuses on identifying and leveraging an individual’s strengths and talents to enhance their performance and overall effectiveness as an executive or leader. Rather than focusing primarily on weaknesses and areas of improvement, this coaching method emphasizes the identification and development of existing strengths and leveraging them to achieve personal and professional goals (MacKie, 2014).

It should be noted that the two approaches outlined above are not mutually exclusive—a coaching session can include elements of both; the distinction is based more on the overarching focus of the coaching and what it is trying to achieve. That said, we were interested in operationalizing this distinction to see if it had an effect on outcomes. Hence, in the present meta-analysis, we attempted to investigate whether one of these overarching approaches was more effective than the other by including the theoretical foundation of coaching (process versus outcome) as a moderator variable.

### 1.3. Coaching outcomes

As with many interventions aimed at improving workplace performance, the question of what outcomes coaching can affect must be answered on several levels. Borrowing from the training effectiveness literature, Kotte and Bozer (2022) described the use of a four-level model based on Kirkpatrick’s hierarchy. The levels in this framework are: subjectively perceived benefit, affective and cognitive learning outcomes, client behavior change, and performance results. In a similar vein, Jones et al. (2016) applied a training-based conceptualization of outcomes presented by Kraiger et al. (1993). This model conceptualizes expected outcomes as falling into three categories: *affective, cognitive and skill based*. Affective outcomes include attitude and motivational outcomes (e.g., self-efficacy, wellbeing). Cognitive outcomes include learning declarative knowledge, problem solving and other cognitive strategies. Finally, skill outcomes include acquisition and automaticity of new skills (e.g., negotiation skills; delegation skills). To this, Jones et al. (2016) added a category called *results* (similar to Kirkpatrick) that represents organizational-level changes and outcomes (e.g., increased sales or lower attrition).

Past research into coaching has employed a variety of effectiveness indicators that represent all levels of the frameworks described above. Unfortunately, there does not seem to be much theoretical concordance between the coaching technique employed and the outcomes assessed. In other words, past researchers have not attempted to draw differential hypothesizes predicting that specific outcomes will be influenced more or less based on the nature of the coaching being studied. For example, it might make theoretical sense to expect affective outcomes to be more affected by facilitation/process-based approaches than outcome/goal setting-based approaches.

In the present meta-analysis we attempted to investigate this question by conducting sub-analyzes that crossed type of outcome by theoretical foundation so that we could assess whether there were differential effects. To do this, we coded the effectiveness outcomes using the same framework as Jones et al. (2016) (i.e., affective, cognitive, skill and results) and looked at two questions. First, was there an overall effect for outcome type and second, did the type of coaching have differential effects on the outcome types.

### 1.4. Previous meta-analyzes

Theeboom et al. (2014) conducted a meta-analytic review of coaching effectiveness in organizations. They concluded that, across a variety of outcomes, coaching had a significant positive effect on individual effectiveness. The effect sizes ranged from *g* = 0.43 for coping to *g* = 0.74 for goal-directed self-regulation. The authors also reported that within-subjects (pre-post only) designs yielded significantly higher effect sizes than mixed designs (pre-post with a comparison group). They also found that the number of coaching sessions was not related to effectiveness.

Jones et al. (2016) critiqued the Theeboom et al. (2014) analysis on the basis that they included studies that were not conducted in the workplace. They also suggested an emphasis on variables that are more relevant to the workplace. In their meta-analysis, they also found that coaching was associated with a moderate positive effect on effectiveness.

Jones et al. (2016) also considered several potential moderator variables. Contrary to Theeboom et al. (2014), Jones et al. (2016) did not find a significant difference between within-subject and mixed research designs. However, like Theeboom et al. (2014), Jones et al. (2016) found no effect of the number of coaching sessions on outcomes.

Additionally, Jones et al. (2016) investigated some potential moderators not considered previously. For example, they reported that internal consultants were significantly more effective than those who were external to the organization. Additionally, they reported no difference between face-to-face versus mixed-modes of delivery. Finally, Jones and her colleagues investigated the impact of multi-source feedback (feedback given not only from a supervisor but also subordinates, peers, clients etc.) in the coaching process. Surprisingly, they found that having multi-source feedback was associated with worse outcomes.

Like Jones et al. (2016), Burt and Talati (2017) sought to improve the Theeboom et al. (2014) analysis. Specifically, they tightened the inclusion criteria to include only pre & post test for treatment and control groups, included unpublished studies and added studies for additional years. They found that, while the overall effect size was somewhat smaller than reported by Theeboom et al. (2014), there was still a moderate positive effect of coaching. There were no moderating effects of age, type of measure, or authors.

### 1.5. The present meta-analysis

Several years have passed since the last meta-analysis of the literature on the effectiveness of workplace coaching. As previously noted, the number of coaches has more than tripled in this timeframe (Passmore and Sinclair, 2020). Moreover, the investment in coaching is estimated at over two billion US dollars a year (International Coaching Federation, 2020). As with many other techniques that promise to improve performance in the workplace, this increase in coaching has been largely uninformed by empirical effectiveness research. And while there is some evidence that coaching can have positive outcomes (Theeboom et al., 2014; Jones et al., 2016), it is not all clear whether some coaching approaches are superior to others, which outcomes are most influenced by coaching or even if coaching can have negative, unintended consequences. Therefore, there is a need to analyze any new data to provide a more current estimate of the effects of coaching and the factors that may influence its effectiveness.

## 2. Hypotheses

*H1*: There will be no difference in coaching effectiveness based on the type of coaching offered (process/facilitation-based or outcome/goal setting-based).

*H2*: There will be no difference between the effectiveness of coaching as assessed by the three types of outcome measures.

*H3*: Studies that employed process/facilitation-based coaching will yield better outcomes for affective-based measures than cognitive or skill-based measures, while the opposite will be true for outcome/goal setting-based coaching.

*H4*: Self-reported outcomes will be higher than either evaluation by supervisors or evaluation by subordinates.

*H5*: Face-to-face coaching will yield better results than virtual coaching.

*H6a*: The duration of coaching as measured by the number of sessions will have a significant positive impact on coaching outcomes.

*H6b*: The duration of coaching as measured by the total hours of coaching will have a significant positive impact on coaching outcomes.

## 3. Methods

### 3.1. Search strategy

A variety of approaches were taken to collect relevant published and unpublished research findings relevant to the meta-analysis. Papers after 2014 (the last meta-analysis) were considered for inclusion. Electronic databases (Web of Science, PsychInfo, JSTOR Business, ERIC, Google Scholar, and ProQuest) were searched using the keywords “coaching,” crossed with “workplace,” “executive,” “effectiveness,” “impact,” and “evaluation.” Additionally, Dissertation Abstracts were searched to seek unpublished studies. Finally, unpublished studies were sought by emailing authors that have been active in the area. No unpublished results were received.

Each article was evaluated for inclusion using the criteria described by Theeboom et al. (2014). Namely, included studies must have been conducted in the workplace by trained coaches. The included studies also needed to provide results regarding work-related outcomes. Included studies needed to include sufficient data to compute an effect size. Finally, only studies that reported individual-level outcomes (as opposed to group or team) were included.

**Figure 1:**
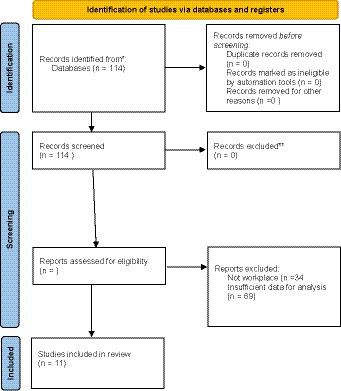
Prisma flow diagram.

The original search yielded a total of 114 papers that were evaluated for inclusion. After evaluating each study against the inclusion criteria, 11 papers were included for the final analysis. These studies are summarized in Table 1. The associated PRISMA flowchart is presented in Figure 1. Several papers were excluded because they did not deal with workplace outcomes. The remainder were excluded because they did not include the data to evaluate coaching outcomes. There were no disagreements among the raters.

**Table 1:** Details about variables and participants of the included studies.

Study	Outcomes	*n*	Hours of Coaching	Coach	Modality	Rater	Coaching Sessions	Outcome Type	Organization	Occupations
Auer et al. (2022)	Authenticity	1,005		External	Virtual	Self		Affective	Unspecified	Unspecified
	Connectedness					Self		Affective		
	Engagement					Self		Affective		
	Life satisfaction					Self		Affective		
	Optimism					Self		Affective		
	Productivity					Self		Affective		
	Resilience					Self		Affective		
De Haan et al. (2019)	Effectiveness coachee	180	12	Internal	Live	Self	12	Skill	Healthcare	Line managers
	Effectiveness manager					Manager		Skill		
Ballesteros-Sánchez et al. (2019)	Cognitive ability	30	12	External	Live	Self	8	Cognitive	Unspecified	Project managers
	Communicating					Self	8	Skill		
	Effectiveness					Self	8	Affective		
	Leading					Self	8	Skill		
	Managing					Self	8	Skill		
	Professionalism					Self	8	Affective		
Peláez et al. (2020)	Engagement	60	6	External	Live	Self	3	Affective	Automotive	Non-supervisory technicians
	Self-rated performance					Self		Skill		
	Supervisor-rated performance					Manager		Skill		
Junker et al. (2021)	Chronic stress	44	6	External	Live	Self	3	Affective	Education	Management students
	Chronic worrying					Self		Affective		
	Goal attainment					Self		Affective		
	Lack of need satisfaction					Self		Affective		
	Stress appraisal					Self		Affective		
	Work demands					Self		Affective		
Onyishi et al. (2021)	Affect balance	151		External	Live	Self		Affective	Police	Officers
	Flourishing					Self		Affective		
	Life satisfaction					Self		Affective		
Williams and Lowman (2018)	Leadership behaviors self	32	4	External	Live	Self	4	Skill	Unspecified	Middle manager or higher
	Leadership behaviors supervisor					Manager	4	Skill		
	Leadership competency self					Self	4	Skill		
	Leadership competency supervisor					Manager	4	Skill		
	Leadership behaviors self		4	External	Live	Self	4	Skill		
	Leadership behaviors supervisor					Manager	4	Skill		
	Leadership competency self					Self	4	Skill		
	Leadership competency supervisor					Manager	4	Skill		
MacKie (2014)	Coaching readiness	30	9	External	Live	Self	6	Affective	Non-Profit	Managers
	Core self-evaluation					Self		Affective		
	Developmental readiness					Self		Affective		
Allan et al. (2018)	Achievement striving	54		External	Live	Self	10	Affective	Unspecified	Unspecified
	Activity					Self		Affective		
	Anxiety					Self		Affective		
	Assertiveness					Self		Affective		
	Competence					Self		Affective		
	Deliberation					Self		Affective		
	Depression					Self		Affective		
	Dutifulness					Self		Affective		
	Excitement					Self		Affective		
	Gregariousness					Self		Affective		
	Hostility					Self		Affective		
	Impulsiveness					Self		Affective		
	Order					Self		Affective		
	Positive emotions					Self		Affective		
	Self-consciousness					Self		Affective		
	Self-discipline					Self		Affective		
	Vulnerability					Self		Affective		
	Warmth					Self		Affective		
Zuberbuhler et al. (2020)	Extra role performance – employees	38	17	External	Live	Other	8	Skill	Automotive	Managers
	Extra role performance – supervisor					Manager		Skill		
	In role performance – employees					Other		Skill		
	In role performance – supervisor					Manager		Skill		
	Leadership skills – employees					Other		Skill		
	Leadership skills – self					Self		Skill		
	Leadership skills – supervisor					Manager		Skill		
	Psychological capital – self					Self		Skill		
	Work engagement – self					Self		Affective		
Steketee et al. (2022)	Autonomy	15		External	Virtual	Self		Affective	Various	Researchers
	Character					Self		Affective		
	Competence					Self		Affective		
	Health					Self		Affective		
	Purpose					Self		Affective		
	Relatedness					Self		Affective		
	Sleep quality					Self		Affective		

### 3.2. Meta-analytic software

We used Comprehensive Meta-Analysis (CMA v4; Borenstein et al., 2022). This software is advantageous in that a) it allows the entry of multiple data formats and b) it allows consideration of both categorical and continuous moderators (through meta-regression).

### 3.3. Calculation of effect sizes

Like Theeboom et al. (2014), we decided that effect sizes based on standardized means were the best fit for this data set, as all included studies reported means and standard deviations and is recommended for small data sets. We also found that the original justification to use the Hedges and Olkin (1984) approach to estimating effect sizes also held for the present data. Namely, it provides a relatively conservative estimate of the lower boundary of the confidence interval (Johnson et al., 1995). Additionally, all estimates were created using the random effects model as recommended by Borenstein et al. (2021). This estimate assumes that the studies are a sample of a larger universe of studies. In so doing, the estimate considers two possible sources of variance: a) within-study error and b) variation of true effects across studies. Again, this is considered to be the more conservative approach as compared to the fixed-effects model (Borenstein et al., 2021).

Heterogeneity was evaluated using Cochran’s *Q* (Cochran, 1954) and the I^2^ statistic (Higgins and Thompson, 2002). This analysis yielded a *Q* value of 126.45 (df = 11, *p* < 0.001), indicating heterogeneity of effect sizes among the studies. The associated I^2^ value was 91.3, indicating that a substantial portion of the variance can be attributed to true effect differences rather than sampling error. However, we noted the presence of a substantial outlier when reviewing the average effect sizes in the individual studies (see Figure 1). Namely, the effect size estimates of Onyishi et al. (2021) were substantially higher than the other included studies (Hedge’s g = 3.52). After removing this study, the *Q* value was reduced to 12.64 (df = 10, *p* > 0.05), indicating a homogeneous pool of effect sizes. Further, the I^2^ was reduced to 20.09, indicating much less variance in the true score estimate. Given that, the subsequent analyzes were conducted without the Onyishi et al. (2021) study as it was a clear outlier.

**Figure 2:**
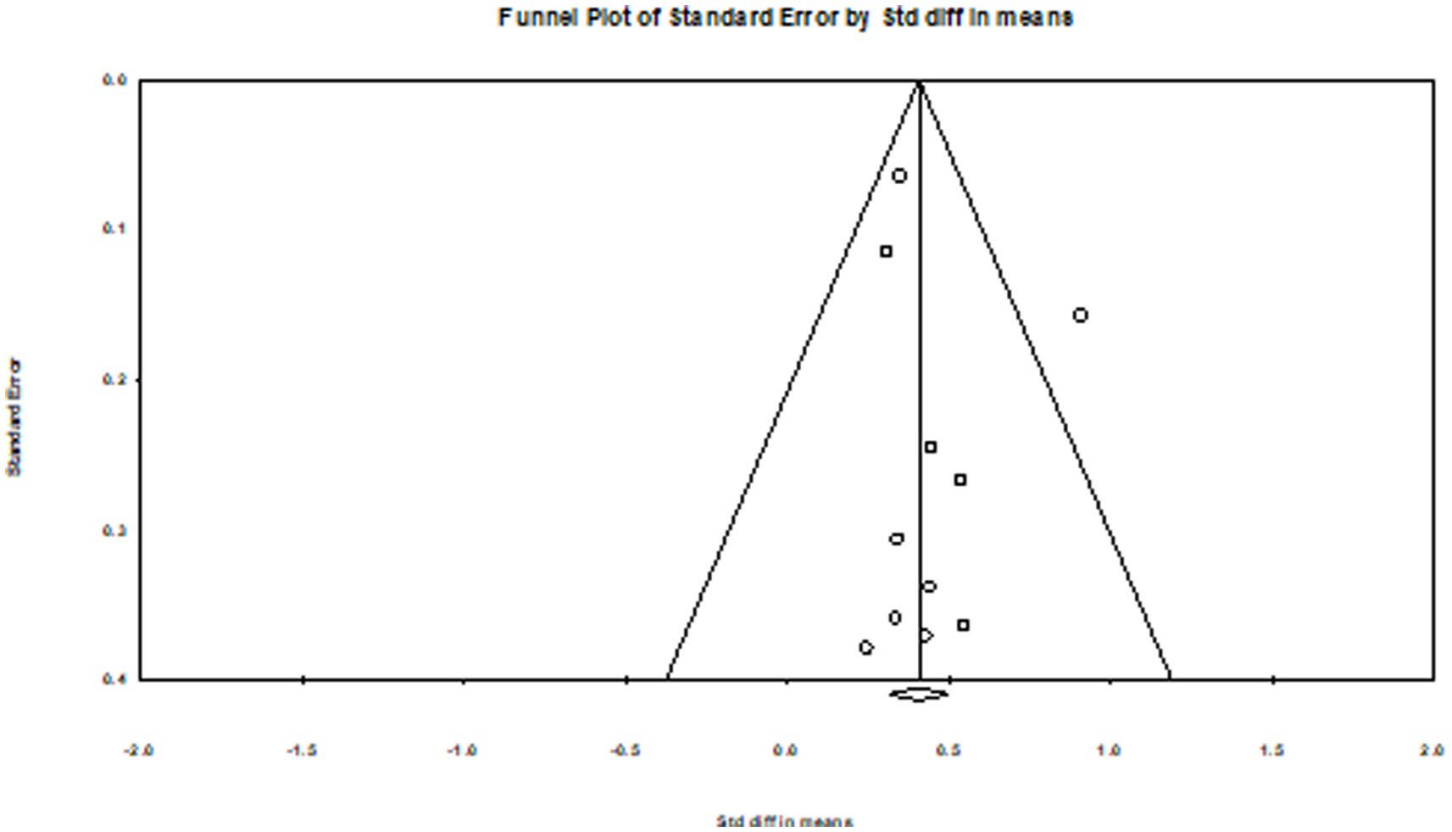
Funnel plot of standardized error by standardized difference in means.

Publication bias was assessed using a funnel plot (see Figure 2). Inspection of the plot suggests that the standard errors were generally symmetrical with regard to the means.

### 3.4. Moderator variables

#### 3.4.1. Theoretical foundation

As noted, we categorized the studies according to whether the coaching approach used a process/facilitation framework or an outcome/goal setting framework by using consensus ratings generated by the authors. The classification ratings were carried out by taking reference to the above-mentioned aspects of the two different theoretical orientations. If the coaching included discussion of achievement of specific outcomes and/or setting of specific goals, we categorized it as outcome/goal. Otherwise, studies that emphasized discussion, identifying obstacles to success and general facilitation by the coach were categorized as process/facilitation. Since there is virtually no literature regarding the relative effectiveness of coaching approaches, we were not able to make a formal prediction about which would yield higher outcomes. Hence, we did not have a hypothesis for this variable.

#### 3.4.2. Outcome type

Individual studies used a variety of outcome measures to assess the effects of coaching. To allow synthesis of these outcomes, we adopted the categorization approach used by Jones et al. (2016) as described above. Outcomes measures were assigned to one of three categories by the first two authors. No discrepancies between the raters occurred. The first category was Affective. This category included attitudinal, emotional, and motivational measures. The cognitive category included outcomes to knowledge, procedures, and strategies. Finally, the skill category refers to measures that involve the development of new skills. None of the included papers reported results that would have fit into the “results” category described by Jones et al. (2016).

We did not have a strong basis for an *a priori* hypothesis regarding the relative expected change in outcomes since previous studies have not shown any differences. We did, however, hypothesize that there would an effect of coaching type on outcomes, such that the process/facilitation-based programs would have a higher impact on affective measures while outcome/goal setting-based programs would yield higher scores on cognitive and skill outcomes.

#### 3.4.3. Evaluation source

Studies of the effectiveness of coaching combine several methods of estimating coaching outcomes. These include self-evaluation, evaluation by supervisors, and evaluation by subordinates. However, previous research has indicated that these assessment sources might yield very different outcomes (*cf.* Dunning, 2013). Therefore, we analyzed the data to determine if the source of the evaluation source moderated coaching outcomes.

#### 3.4.4. Modality

Several studies have reported that technology-based communication may be substantially different than face-to-face communication (see Walther et al., 2015 for a review). It is reasonable to hypothesize that the differences may result in different coaching outcomes depending on modality. Therefore, we analyzed face-to-face versus virtual modalities as a possible moderator variable.

#### 3.4.5. Number of sessions/hours of coaching

Previous meta-analyzes have demonstrated that the amount of coaching was not related to coaching outcomes. This runs counter to the traditional dose–response relationship that one might expect in areas such as psychotherapy. Due to the variability in past coaching practice, it is possible that the correlation between the amount of coaching and coaching outcomes was obscured. Since modern coaches are more likely to have had formal training than in the past and it can be expected that the coaching practices are more oriented to certain standards, it might be that this hypothesis is now supported.

## 4. Results

### 4.1. Aggregated effect sizes

The weighted effect sizes, averaged across outcomes is presented in Table 2. As illustrated in the Table, the point estimate was significant. This result indicates that, across studies and outcome measures, coaching interventions are likely to have a medium positive effect. Although the I^2^ was relatively low [after excluding the Onyishi et al. (2021) study], we evaluated select moderator variables in the service of replicating and extending the existing meta-analyzes. The details of this analysis are presented in Table 3.

**Table 2:** Aggregated and weighted effect sizes, averaged across the outcomes for each included study.

Model	Study name	Outcome	Statistics for each study						
			Hedges’s g	Standard error	Variance	Lower limit	Upper limit	Z-Value	*p*-Value
	Auer 2022	Combined*	0.346	0.064	0.004	0.220	0.471	5.400	0.000
	De Haan	Combined*	0.902	0.156	0.024	0.596	1.208	5.781	0.000
	Ballesteros-Santos	Combined*	0.415	0.360	0.130	−0.291	1.120	1.152	0.249
	Pelaez 2019	Combined*	0.528	0.263	0.069	0.012	1.043	2.007	0.045
	Junker 2021	Combined*	0.333	0.300	0.090	−0.255	0.922	1.110	0.267
	Williams 2018 Goal	Combined*	0.324	0.349	0.122	−0.359	1.008	0.930	0.353
	Williams 2018 Process	Combined*	0.530	0.354	0.125	−0.163	1.223	1.498	0.134
	Mackie 2015	Combined*	0.236	0.368	0.135	−0.485	0.957	0.642	0.521
	Allan 2018	Combined*	0.295	0.111	0.012	0.078	0.513	2.662	0.008
	Pelaez 2020b	Combined*	0.428	0.330	0.109	−0.219	1.075	1.295	0.195
	Stekehee 2022	Combined*	0.418	0.232	0.054	−0.035	0.872	1.807	0.071
Random			0.430	0.066	0.004	0.301	0.558	6.532	0.000
Pred Int			0.430			0.170	0.689		

*Combined indicated that multiple outcomes were aggregated.

**Table 3:** Results of the meta analyzes with reference to aggregated effect sizes and the moderator factors.

Variable	*k*	Hedge’s *g*	CI	Z	*p*
*Aggregate*	11	0.44	0.31–0.57	6.53	0
*Theoretical foundation*					
Process-oriented	2	0.45	0.07–0.83	2.33	0.02
Outcome-oriented	6	0.39	0.14–0.65	3	0.003
*Outcome type*					
Skill	5	0.72	0.49–0.95	6.03	0
Affective	8	0.41	0.26–0.58	3.01	0.003
*Coaching type × Outcome type*					
Process/Skill	1	0.53	−0.16–1.12	1.5	0.13
Outcome/Skill	4	0.42	0.10–0.72	2.62	0.009
Process/Affective	1	0.42	−0.04–0.85	1.81	0.07
Outcome/Affective	5	0.48	0.20–0.76	3.37	0.001
*Evaluation source*					
Manager	5	0.5	0.09–0.9	2.42	0.002
Self	6	0.41	0.31–0.51	3.89	0
Subordinate	1	0.24	−0.4–0.88	0.74	0.461
*Modality*					
Face-to-Face	9	0.48	0.29–0.67	2.72	0.007
Virtual	2	0.35	0.23–0.47	5.69	0

### 4.2. Moderating effect of theoretical foundation

*H1*: There will no difference in coaching effectiveness based on the type of coaching offered (process/facilitation-based or outcome/goal setting-based).

Theoretical approach (process vs. outcome) was analyzed as a moderator variable. There was no significant difference between the two approaches. Process-oriented coaching yielded a point estimate of g = 0.45 while Outcome-oriented coaching indicated a point estimate of 0.39. The details of this analysis are presented in Table 3.

### 4.3. Moderating effect of outcome type

*H2*: There will be no difference between the effectiveness of coaching as assessed by the three types of outcome measures.

To test this hypothesis, we evaluated the degree to which reported outcomes vary as a result of the type of outcome by computing effect sizes separately for each outcome type. Outcomes related to skill yielded a significant point estimate of g = 0.72. Affective outcomes yielded a point estimate of *g* = 0.41. The effect sizes were not significantly different. The details of this analysis are presented in Table 3. None of the studies assessed cognitive outcomes according to the definition outlined above.

### 4.4. Effects of coaching type on outcome type

*H3*: Studies that employed process/facilitation-based coaching will yield better outcomes for affective-based measures than cognitive or skill-based measures, while the opposite will be true for outcome/goal setting-based coaching.

To assess the hypothesis that different types of coaching would create different outcomes, we conducted separate analyzes on the skill and affective outcome types using coaching type as a moderator. When considering Affective outcomes, process-based coaching yielded an effect estimate of g = 0.42. Outcome-based coaching yielded a similar point estimate (g = 0.48). The details of this analysis are presented in Table 3.

Looking at skill outcomes, process-based coaching was associated with a point estimate of 0.53. However, this was not significant, likely due to the small sample size. Outcome-based coaching yielded a point estimate of 0.42. The details of this analysis are presented in Table 3.

Due to the limited number of studies, we were only able to evaluate Hypothesis 3 for outcome based coaching. The results indicate no significant difference in creating skill versus affective outcomes (Z_Diff_ = 0.77, *p* = n.s.).

### 4.5. Moderating effect of evaluation source

*H4*: Self-reported outcomes will be higher than either evaluation by supervisors or evaluation by subordinates.

The results of our analysis of this hypothesis indicated that positive outcomes were reported regardless of evaluation source. The highest point estimate resulted from manager-rated outcomes (g = 0.50). Self-reported outcomes were also associated with positive estimates (*g* = 0.41). However, the one study that investigated evaluations from subordinates yielded a smaller positive, but not significant, point estimate (*g* = 0.24). This estimate was significantly lower than the other two categories (Q(2) = 0.39, *p* > 0.05). Again, however, since the employee-rated effect size was based on only one study, it may change as more studies investigate this effect. The details of this analysis are presented in Table 3.

### 4.6. Moderating effect of modality

*H5*: Face-to-face coaching will yield better results than virtual coaching.

To examine this hypothesis, we explored the differences in face-to-face versus virtual modalities in coaching outcomes. The results indicate that both face-to-face and virtual coaching were associated with significant positive outcomes. Face-to face coaching yielded a point estimate of *g* = 0.48, Virtual coaching yielded a point estimate of *g* = 0.35. The difference between the two groups was not significant (Q(1) = 0.67, *p* > 0.05). The details of this analysis are presented in Table 3.

### 4.7. Moderating effect of number of sessions

*H6a*: The duration of coaching as measured by the number of sessions will have a significant positive impact on coaching outcomes.

To test this hypothesis, a meta-regression was used to evaluate whether number of sessions (*k* = 9) was related to coaching outcomes. The result of this analysis indicates that the number of sessions was not a significant predictor of overall coaching outcomes (Z = 1.03, *p* = 0.30).

### 4.8. Moderating effect of hours of coaching

*H6b*: The duration of coaching as measured by the total hours of coaching will have a significant positive impact on coaching outcomes.

To test this hypothesis, a meta-regression was used to evaluate whether total hours of coaching (*k* = 8) was related to coaching outcomes. The result of this analysis indicates that the number of sessions was not a significant predictor of overall coaching outcomes (Z = 0.1.15, *p* = 0.25).

## 5. Discussion

There has been an explosion in the popularity and use of workplace coaching since the last published meta-analysis, with considerable resources (time and money) invested in it. The goal of the present study was to evaluate how coaching has evolved and whether there has been a change in its estimated impact or a better understanding of the variables that might moderate its effectiveness. We hoped that the results of this study could shed light on the best approaches for training coaches and guidance for optimizing the delivery of coaching in the workplace. While we partially accomplished this goal, perhaps the strongest contribution of this work is highlighting what still needs to be done. We begin by reviewing what we found (in this section) and then turn our discussion toward outlining what we believe is needed to move the field forward.

After removing one clear outlier, the overall effect of coaching was positive and of moderate effect size. Interestingly, this effect was relatively homogenous (Q = 12.64) as compared to past analyzes. Similar to past analyzes (Theeboom et al., 2014; Jones et al., 2016), this finding was also positive across all outcome types. That is, there was no difference in the effectiveness of coaching as a function of which outcome measure was used. Overall, based on three meta-analyzes (representing thousands of data points) it is safe to conclude that coaching is an effective workplace intervention.

That said, we were interested in seeing whether we could meaningfully distinguish different types of coaching based on the theoretical framework upon which they were developed. The data did not yield positive results in this regard; indeed, there was no difference between coaching that stemmed from a process/facilitation framework versus an outcome/goal setting framework. There are several reasons this might be the case.

First, the level of homogeneity just mentioned was so high, that there was insufficient variance to detect a difference given the sample size. Second, the two approaches might actually be equally effective. Or third—and most likely—the approaches are not sufficiently differentiated to allow meaningful comparisons. Unfortunately, the vast majority of studies lacked enough detail to make clear determinations.

For likely similar reasons, we did not find the hypothesized relationship between coaching type and outcome type. Specifically, process/facilitation-based coaching did not have a greater impact on affective outcomes nor did the outcome/goal setting-based coaching have a greater impact on skill outcomes. Given the level of detail in the studies, it is impossible to know why this was the case for the reasons noted above.

With respect to the modality of coaching, the COVID-19 pandemic heightened interest in the effectiveness of virtual coaching. Therefore, we considered the differences between virtual and live coaching. Our analysis revealed no significant difference between the two modalities, supporting the assertion that virtual coaching can be a useful tool in effecting workplace effectiveness. This is consistent with Jones et al.’s finding about hybrid coaching. It is an important finding because it gives remote coaches confidence that coaching can be as effective if carried out virtually as it is in face-to-face interactions. This profoundly increases the possible pool of both coaches and clients and could help coaching become even more popular in the future.

Like previous analyzes, we also investigated the moderating effect of the number of coaching sessions. Also like the previous analyzes, we found this variable not to be a significant moderator of coaching outcomes. Because we were concerned that studies “sessions” are of varying length, we also considered the total number of hours of coaching received. Again, this effect was not significant. Besides being durable, this is an interesting finding that requires further consideration. On the one hand, it could reflect a sort of demand characteristic where the act of being coached (independent of the actual coaching) is responsible for the positive outcomes. This seems unlikely (McCambridge et al., 2012), but given the current data, it is feasible. More likely, the number of sessions required to achieve desired outcomes varies considerably across studies.

Finally, we considered the moderating impact of the source of evaluator used to judge the perceived impact of coaching. The results indicated that both self-reports and supervisor reports yielded moderate, positive, and significant point estimates. However, the one study that reported results based on employee (subordinate) ratings yielded a negative, but not significant, estimate. This is an issue that requires additional investigation in future studies of coaching effectiveness.

### 5.1. Directions for future research

Including the present study, reviews and meta-analyzes have been consistent in reporting a moderate, positive effect of coaching for over a decade. This effect seems robust across outcomes, number of sessions, and modality—clearly something is working! The question now becomes, how can coaching outcomes be optimized? We attempted to begin answering this question by investigating whether the theoretical bases of coaching could determine which approaches are more successful than others. We were unable to do this, and it is unclear why the distinctions we drew were not meaningful.

Our strongest conclusion from this exercise is that future researchers would do the field a great service by including details about the coaching approach when publishing studies. This would allow us to better understand the similarities and differences between approaches so that they can be better associated with specific outcomes. In fact, the *science of coaching* might benefit from a taxonomy of standardized coaching approaches, strategies, and techniques to assist us in better understanding which interventions are best for any given client, situation and/or desired outcome.

Another set of observations pertains to the outcome measures themselves. While we are not advocating that the Kraiger et al. (1993) categorization of outcomes, or one based on Kirkpatrick’s hierarchy, or any other is superior, we are suggesting that some theoretical framework of outcome types be used when studies are conceived. Indeed, the field would be well served by thinking through which outcomes are expected to be affected by coaching in general and which are expected to change due to specific elements or approaches of coaching in particular. In addition, like Jones et al., none of the studies included in our analysis used outcomes as the results level. At this point in the history of coaching as an organizational intervention, it is important for research to establish positive influences on organizational results as a function of coaching. Without this, it will be impossible to conduct cost–benefit analyzes to justify continued investment in coaching.

Related to the question of what is the best type of coaching is the related question of, “who are the best coaches?” According to ICF, managers and leaders using coaching skills strongly agree that clients expect coaches to be certified and/or credentialed. However, the studies represented in this analysis include coaches with a very wide range of backgrounds and experience. Interestingly, very few studies include information about the coach’s certification (ICF or otherwise). Without such information, it is difficult to associate the quality of coaches with the outcomes they create.

Likewise, studies of coaching include very little information about the nature of coaching clients. For the most part, the studies reported here used volunteers in large organizations as the clients. However, this may not generalize well to the actual clients who seek out or are offered coaching. Almost nothing is reported about the client’s coaching goals or previous experiences. So, while it’s valuable that coaching “can” create good outcomes (as defined by the researcher), it would be better to demonstrate that coaching can help clients achieve *their* goals. This may represent the more externally valid outcome. Hence, we recommend that future research into coaching effectiveness take a more client-centric view, specifically reporting whether outcomes were consistent with the client’s goals and desires.

Another observation regarding passed studies into coaching effectiveness is that they have focused on an undifferentiated (or at least undefined) set of desired changes. However, there is a trend in the industry towards coaching designed to address a specific set of skills and outcomes--e.g., conflict resolution (Brinkert, 2016) or leadership (Wise and Hammack, 2011). This is another area where additional outcome research may provide more targeted guidance to the coaching community.

In another vein, a viable question that remains unanswered is “how many coaching sessions (or hours) are needed to see results?.” All three meta-analyzes have found that the number of sessions/h does not predict outcomes. As noted, this could be some sort of demand characteristic, but is more likely due to the coarseness of the data. Future researchers would do well to adopt more precise measurement schemes that can track outcomes over the course of a coaching relationship (e.g., longitudinal, within-subjects designs). This would reveal when outcomes are changing and help answer the question of how much coaching is needed. It might also uncover important individual differences in coaching effectiveness.

The issue of unwanted effects stemming from coaching also requires further study. Schermuly and Graßmann (2019) presents a number of possible side effects that could possibly occur. These include relationship problems with supervisors, dependence on the coach, and possible reduction in job satisfaction. At this point, it is unknown how prevalent these—or other—unwanted effects occur as a function of coaching. This should be the subject of future research.

Finally, scholars in the area of workplace coaching have repeatedly called for more theory-focused research (i.e., Theeboom et al., 2014; Bozer and Jones, 2021). Ideally, scientists would advance theories that propose a “mechanism of action” for specific coaching outcomes. Examples could include goal setting, appreciative inquiry, cognitive-behavioral approaches, positive psychology, and others (Sutton, 2020). Empirical research could then test these mechanisms with the goal of identifying the specific coaching activities that could support the client’s goal. However, researchers in this area have been slow to adopt this approach. There is a clear need for additional theoretical work to support the explosion of interest in workplace coaching and to guide future research.

### 5.2. Limitations

Despite the increase in interest regarding workplace coaching, the empirical literature in this area is still quite limited. As noted above, there is a lack of detail about the coaches, the coachees, and the content of coaching sessions. Furthermore, there is often a lack of detail regarding the setting in which the coaching was provided. This makes it difficult to build a knowledge base in any meaningful way. Furthermore, there is simply a lack of controlled studies. As noted in the results, some moderator variables only included one study, limiting the confidence one can have in the conclusions. Finally, several studies did not include adequate statistical data to allow inclusion in the meta-analysis. It would be helpful if journals in this area enforced for stringent requirements for reporting results.

### 5.3. Recommendations for advancing the *science of coaching*

Consistent with two previous meta-analyzes, our analysis found that, overall, coaching is an effective intervention for improving workplace outcomes. At this point, in order to establish and advance a *science of coaching*, we recommend the following:

Future researchers need to include details on the type of coaching approach being followed. As noted, ultimately, a taxonomic approach that defines various approaches and their attributes is desirable. For the moment, researchers need to at least describe the approach in enough detail that readers understand the way the coaching was carried out.
Future research needs to better explicate the types of outcomes that can be expected from coaching and also attempt to associate specific coaching approaches and features with expected outcomes. Further, longer term, results-level outcomes of coaching need to be investigated more often.
Future research would benefit from specification of the coach’s credentials. While there seems to be a desire on the part of clients for coaches to have credentials/certifications, an empirical look at the relationship between these and effectiveness would be useful. For example, the results of such analyzes could inform the manner in which coaches are trained.
Future research should employ actual coaching clients or, if volunteers are used, at least define better who the clients are.
Future researchers need to consider whether coaching is effective in achieving more targeted outcomes (e.g., improved conflict resolution skills) as well as more generic ones.
Future researchers should consider longitudinal, within-studies designs that track outcomes more precisely over time.
Future research needs to be more theoretically grounded and strive to better understand the “mechanisms of action” of coaching. This is related to the first recommendation focusing on coaching approaches but goes further by seeking to understand the specific aspects of the coaching relationship that can account for desired outcomes. Findings from such studies can inform the development of more effective and possibly efficient coaching strategies.

## Data availability statement

The original contributions presented in the study are included in the article/supplementary materials, further inquiries can be directed to the corresponding author.

## Author contributions

JC-B and CB were responsible for conceptualizing this research project and conducting all analyzes. CC, SD, JE, and JH were responsible for identifying relevant articles, evaluating articles against inclusion criteria and coding moderator variables. All authors contributed to the article and approved the submitted version.

## References

[ref1] * indicates inclusion in the analysis.

[ref2] *Allan J. Leeson P. Martin S. (2018). Application of a 10 week coaching program designed to facilitate volitional personality change: Overall effects on personality and the impact of targeting. Int. J. Evid. Based Coach. Mentor., 16, 80–94, doi: 10.24384/000470

[ref3] *Auer E. M. Hutchinson D. Eatough E. Carr E. W. Sinar E. F. Kellerman G. (2022). The buffering effects of virtual coachingduring crisis: A quasi-experimental study ofchanges in well-being, work, and socialoutcomes before and during the COVID-19pandemic. Int. J. Evid. Based Coach. Mentor., 20 3–19, doi: 10.24384/ektn-xx15

[ref4] Ballesteros-Sánchez L. Ortiz-Marcos I. Rodríguez-Rivero R. (2019). The impact of executive coaching on project managers’ personal competencies. Proj. Manag. J. *50*, 306–321. doi: 10.1177/8756972819832191, PMID: 37219882

[ref5] Bono J. E. Purvanova R. K. Towler A. J. Peterson D. B. (2009). A survey of executive coaching practices. Pers. Psychol. 62, 361–404. doi: 10.1111/j.1744-6570.2009.01142.x, PMID: 36108091

[ref6] Borenstein M. Hedges L. E. Higgins J. P. T. Rothstein H. R. (2022). Comprehensive Meta-Analysis Version 4. In Biostat, Inc. Available at: www.Meta-Analysis.com

[ref7] Borenstein M. Hedges L. V. Higgins J. P. Rothstein H. R. (2021). Introduction to meta-analysis. John Wiley & Sons. Hoboken, NJ.

[ref8] Bozer G. Delegach M. (2019). Bringing context to workplace coaching: A theoretical framework based on uncertainty avoidance and regulatory focus. Hum. Resour. Dev. Rev. *18*, 376–402. doi: 10.1177/1534484319853098

[ref9] Bozer G. Jones R. J. (2021). Introduction to the special issue on advances in the psychology of workplace coaching. Appl. Psychol. *70*, 411–419. doi: 10.1111/apps.12305

[ref10] Brinkert R. (2016). State of knowledge: Conflict coaching theory, application, and research. Confl. Resolut. Q. *33*, 383–401. doi: 10.1002/crq.21162, PMID: 26229119

[ref11] Burt D. Talati Z. (2017). The unsolved value of executive coaching: A meta-analysis of outcomes using randomised control trial studies. Int. J. Evid. Based Coach. Mentor. 15, 17–24. doi: 10.24384/000248

[ref12] Cochran W. G. (1954). The combination of estimates from different experiments. Biometrics 10, 101–129. doi: 10.2307/3001666, PMID: 37735349

[ref13] *De Haan E. Gray D. E. Bonneywell S. (2019). Executive coaching outcome research in a field setting: A near-randomized controlled trial study in a global healthcare corporation. Acad. Manag. Learn. Edu., 18, 581–605, doi: 10.5465/amle.2018.0158

[ref14] Dunning D. (2013). “The problem of recognizing one’s own incompetence: Implications for self-assessment and development in the workplace” in Judgment and decision making at work. eds. Highhouse S. Dalal R. Salas E. (England: Routledge), 57–76.

[ref15] Freire T. (2013). “Positive psychology approaches” in The Wiley-Blackwell handbook of the psychology of coaching and mentoring. eds. Passmore J. Peterson D. B. Freire T. (Hoboken, NJ: John Wiley & Sons), 426–442.

[ref16] Grant A. M. (2003). The impact of life coaching on goal attainment, metacognition and mental health. Soc. Behav. Personal. Int. J. 31, 253–264. doi: 10.2224/sbp.2003.31.3.253

[ref17] Grant A. M. (2022). “Solution-focused coaching: The basics for advanced practitioners” in Coaching Practiced. eds. Tee D. Passmore J. (Hoboken, NJ: Wiley), 311–322.

[ref18] Grant A. M. Cavanagh M. J. (2007). Evidence-based coaching: Flourishing or languishing? Aust. Psychol. *42*, 239–254. doi: 10.1080/00050060701648175, PMID: 37667829

[ref19] Greif S. Möller H. Scholl W. Passmore J. Müller F. (2022). “Coaching definitions and concepts” in International handbook of evidence-based coaching: theory, research and practice. eds. Greif S. Möller H. Scholl W. Passmore J. Müller F. (New York: Springer International Publishing), 1–12.

[ref20] Hedges L. V. Olkin I. (1984). Nonparametric estimators of effect size in meta-analysis. Psychol. Bull. *96*:573. doi: 10.1037/0033-2909.96.3.573, PMID: 35112575

[ref21] Higgins J. P. Thompson S. G. (2002). Quantifying heterogeneity in a meta-analysis. Stat. Med. *21*, 1539–1558. doi: 10.1002/sim.1186, PMID: 12111919

[ref22] International Coaching Federation (2020). Global Coaching Study. Available at: https://coachingfederation.org/research/global-coaching-study

[ref23] Johnson B. T. Mullen B. Salas E. (1995). Comparison of three major meta-analytic approaches. J. Appl. Psychol. *80*:94. doi: 10.1037/0021-9010.80.1.94, PMID: 34644040

[ref24] Jones R. J. Woods S. A. Guillaume Y. R. (2016). The effectiveness of workplace coaching: A meta-analysis of learning and performance outcomes from coaching. J. Occup. Organ. Psychol. *89*, 249–277. doi: 10.1111/joop.12119

[ref25] *Junker S. Pömmer M. Traut-Mattausch E. (2021). The impact of cognitive-behavioural stress management coaching on changes in cognitive appraisal and the stress response: A field experiment. Coach. Int. J. Theory Res. Pra., 48, 134–144, doi: 10.1080/17521882.2020.1831563

[ref9001] Kilburg R. R. (1996). Toward a conceptual understanding and definition of executive coaching. Consult. Psychol. J. Pract. Res. 62, 361–404. doi: 10.1037/1061-4087.48.2.134

[ref26] Kotte S. Bozer G. (2022). “Workplace coaching research: Charted and uncharted territories” in International Handbook of Evidence-Based Coaching. eds. Siegfried G. Möller H. Scholl W. Passmore J. Müller F. (Cham: Springer), 971–982.

[ref27] Kraiger K. Ford J. K. Salas E. (1993). Application of cognitive, skill-based, and affective theories of learning outcomes to new methods of training evaluation. J. Appl. Psychol. *78*:311. doi: 10.1037/0021-9010.78.2.311

[ref28] *MacKie D. (2014). The effectiveness of strength-based executive coaching in enhancing full range leadership development: A controlled study. Consult. Psychol. J., *66*,:118, doi: 10.1037/cpb0000005

[ref29] McCambridge J. de Bruin M. Witton J. (2012). The Effects of Demand Characteristics on Research Participant Behaviours in Non-Laboratory Settings: A Systematic Review. PLoS One 7:e39116. doi: 10.1371/journal.pone.0039116, PMID: 22723942 PMC3378517

[ref30] Möeller H. Kotte S. (2022). “Assessment in coaching” in International handbook of evidence-based coaching: theory, research and practice. eds. Greif S. Möller H. Scholl W. Passmore J. Müller F. (Cham: Springer International Publishing), 55–64.

[ref31] *Onyishi C. N. Ede M. O. Ossai O. V. Ugwuanyi C. S. (2021). Rational emotive occupational health coaching in the management of police subjective well-being and work ability: a case of repeated measures. J. Police Crim. Psychol., *36*, 96–111, doi: 10.1007/s11896-019-09357-y

[ref32] Passmore J. Sinclair T. (2020). Becoming a Coach: The Essential ICF Guide. Springer, New York.

[ref33] Passmore J. Peterson D. Freire T. (2013). “The psychology of coaching and mentoring” in The Wiley-Blackwell handbook of the psychology of coaching and mentoring. eds. Passmore J. Peterson D. B. Freire T. (Hoboken, NJ: Wiley), 1–11.

[ref34] *Peláez M. J. Coo C. Salanova M. (2020). Facilitating work engagement and performance through strengths-based micro-coaching: a controlled trial study. J. Happiness Stud., *21*, 1265–1284. doi: 10.1007/s10902-019-00127-5

[ref35] Schermuly C. C. Graßmann C. (2019). A literature review on negative effects of coaching–what we know and what we need to know. Coach. Int. J. Theory Res. Pra. *12*, 39–66. doi: 10.1080/17521882.2018.1528621

[ref36] Sherman S. Freas A. (2004). The wild west of executive coaching. Harv. Bus. Rev. *82*, 82–90. PMID: 15559448

[ref37] Silzer R. Church A. Rotolo C. Scott J. (2016). I-O Practice in Action: Solving the Leadership Potential Identification Challenge in Organizations. Ind. Organ. Psychol. *9*, 814–830. doi: 10.1017/iop.2016.75

[ref38] Sonesh S. C. Coultas C. W. Marlow S. L. Lacerenza C. N. Reyes D. Salas E. (2015). Coaching in the wild: Identifying factors that lead to success. Consult. Psychol. J. *67*:189. doi: 10.1037/cpb0000042

[ref39] *Steketee A. Chen S. Nelson R. A. Kraak V. I. Harden S. M. (2022). A mixed-methods study to test a tailored coaching program for health researchers to manage stress and achieve work-life balance. Transl. Behav. Med., *12*, 369–410, doi: 10.1093/tbm/ibab134, PMID: 34718809

[ref40] Sutton J. (2020) Coaching styles explained: 4 different approaches. Available at: Positivepsychology.com

[ref41] Theeboom T. Beersma B. van Vianen A. E. (2014). Does coaching work? A meta-analysis on the effects of coaching on individual level outcomes in an organizational context. J. Posit. Psychol. *9*, 1–18. doi: 10.1080/17439760.2013.837499

[ref42] Vandaveer V. V. Lowman R. L. Pearlman K. Brannick J. P. (2016). A practice analysis of coaching psychology: Toward a foundational competency model. Consult. Psychol. J. *68*, 118–142. doi: 10.1037/cpb0000057

[ref43] Walther J. B. Van Der Heide B. Ramirez A. Jr. Burgoon J. K. Peña J. (2015). “Interpersonal and hyperpersonal dimensions of computer-mediated communication” in The handbook of the psychology of communication technology. ed. Sunhar S. (Hoboken, NJ: Wiley), 1–22.

[ref44] Whitmore J. (2010). Coaching for performance: growing human potential and purpose: the principles and practice of coaching and leadership. Hachette UK. Edinburgh

[ref45] *Williams J. S. Lowman R. L. (2018). The efficacy of executive coaching: An empirical investigation of two approaches using random assignment and a switching-replications design. Consult. Psychol. J. Pract. Res., *70*,:227, doi: 10.1037/cpb0000115

[ref46] Wise D. Hammack M. (2011). Leadership coaching: Coaching competencies and best practices. J. Sch. Leadersh. *21*, 449–477. doi: 10.1177/105268461102100306, PMID: 37577254

[ref47] *Zuberbuhler M. J. Salanova M. Martínez I. M. (2020). Coaching-based leadership intervention program: A controlled trial study. Front. Psychol., 10:3066, doi: 10.3389/fpsyg.2019.03066 32116873 PMC7011779

